# Strength and Impact Toughness of Multilayered 7075/1060 Aluminum Alloy Composite Laminates Prepared by Hot Rolling and Subsequent Heat Treatment

**DOI:** 10.3390/ma19010062

**Published:** 2025-12-23

**Authors:** Hui Zhang, Shida Liu, Siqi He, Qunjiao Wang, Fuguan Cong, Yunlong Zhang, Yu Cao

**Affiliations:** 1 Key Laboratory of Electromagnetic Processing of Materials (Ministry of Education), Northeastern University, Shenyang 110819, China; liushida012@163.com (S.L.); h19792002@163.com (S.H.); wang-qunjiao@epm.neu.edu.cn (Q.W.); 2 Northeast Light Alloy Co., Ltd., Harbin 150060, China; cfgdy2010@163.com (F.C.); zyl030202@126.com (Y.Z.); 3 College of Materials Science and Engineering, Chongqing University, Chongqing 400044, China; yucao928@cqu.edu.cn

**Keywords:** roll bonding, laminates, delamination, strength, toughness

## Abstract

The roll bonding of 7075/1060 composite laminates offers a promising approach toward the increase in toughness of aluminum layered composites. In this paper, 7075 and 1060 aluminum alloy plates were hot roll bonded to fabricate multilayered composite laminates. Solid solution at 470 °C for different holding times and subsequent aging were carried out for all the laminates. This study investigated the effect of holding times on the interfacial microstructure and interfacial bonding strength of the laminates. The interfacial shear strength was found to increase with longer holding times, which was attributed to the solid solution strengthening of the 1060 layer resulting from element diffusion. The findings also reveal that both tensile strength and toughness are positively correlated with the holding time of the solid solution, and there is a simultaneous improvement of tensile strength and toughness as the holding time increases. Microstructural characterization of the crack path profile of the Charpy impact and bending test indicates that interfacial delamination and main crack deflection become pronounced with the increase in holding time, and these lead to an increase in the fracture resistance in the crack-arrester orientation.

## 1. Introduction

Improving the strength and toughness of lightweight metallic materials has consistently been a technical challenge in structural materials research. Laminate metal composites (LMCs) are a kind of heterogeneous metallic material [1,2]. These materials typically comprise alternating layers of high-strength and ductile metals, where the former provides strength and the latter offers considerable deformation capacity. Metallic materials with architecturally designed heterogeneous microstructures have shown the potential to overcome the strength–ductility trade-off to some extent. Studies have demonstrated that layered composite configurations, including bilayer, trilayer, and multilayer structures, can achieve superior combinations of strength and toughness [3,4,5,6,7]. In a study on the tensile strength and fracture of roll-bonded Cu/Al/Cu trilayer laminates, researchers found that over the range of rolling reductions and annealing temperatures investigated, the composites maintained high strength while achieving superior elongation compared to their constituent metals [8,9]. This led to the development of a processing strategy that utilizes controlled interfacial bonding strength to produce laminated composites with enhanced ductility without any significant loss in strength. Extensive research has demonstrated that metallic-laminated composites possess both high strength and remarkable resistance to crack propagation. Furthermore, the toughening mechanism achieved through controlled delamination has also been observed in monolithic steel materials [10], hot-rolled steel clad plates [11], aluminum alloy clad plates [12,13,14], and copper alloy clad plates [15]. These monolithic and clad plates exhibit a desirable combination of high strength and considerable toughness. The enhanced toughness is primarily attributed to energy dissipation through interfacial delamination at multiple interfaces, which accompanies the propagation of the main crack during fracture.

7xxx series aluminum alloys, strengthened by age hardening, offer ultra-high strength and moderate elongation, making them widely applicable in aerospace and aviation industries. Furthermore, the easy availability of 7xxx series sheets and mature rolling technology make the resulting roll-bonded clad plates cost-effective.

Previous studies have demonstrated that hot-rolled multilayer clad plates, such as 7xxx/1xxx and other configurations, can be fabricated by controlling rolling parameters such as reduction ratio and passes [16,17]. These composites achieve high toughness through extrinsic toughening mechanisms, primarily interfacial delamination. This mechanism highlights the critical role of both interfacial bonding strength and the inherent toughness of the constituent layers in determining the overall toughness of the clad plate.

In this study, a 7075 aluminum alloy plate serves as the ultra-high-strength constituent to provide the primary load-bearing capacity, while AA1060 foil, a highly ductile aluminum alloy, acts as the bonding interlayer to facilitate roll bonding. Following roll bonding, a solution treatment and aging are applied to enhance the overall strength of the composite and to tailor the interfacial bonding properties. The relationship between strength and toughness is systematically investigated through uniaxial tension, three-point bending and Charpy impact tests.

## 2. Materials and Methods

### 2.1. Sample Preparation

The materials used were commercially rolled pure Al foils (1060, with a purity of 99.6%, wt. %) with an initial thickness of 0.1 mm and commercially rolled 7075 plates in the T6 temper condition with an initial thickness of 2 mm. The chemical compositions of the 7075 aluminum alloy are presented in Table 1. Tensile tests were performed on the as-received 7075 plate, and the yield strength (YS), ultimate tensile strength (UTS) and elongation are summarized in Table 2.

The 1060 and 7075 alloy plates were cut into rectangles with dimensions of 65 mm (width) × 150 mm (length) along the rolling direction and then annealed at 450 °C for 60 min. The surfaces of the plates to be joined were processed by degreasing in acetone and then scratch brushing with a wire brush. The multilayered composite laminate was prepared by alternately laminating ten layers of 7075 and nine layers of 1060 with 7075 plates positioned as the outermost layers. The leading edge of this 7075/1060 stack was riveted together to ensure integrity during rolling. Prior to rolling, the assembled packet was heated at 450 °C in an air furnace for 30 min. The roll bonding was performed using a laboratory-scale two-high mill with a roll diameter of 450 mm. Roll bonding was achieved through two rolling passes with rolling reductions of 30% and 20%, respectively. Subsequently, the rolled composite laminate was cooled to room temperature.

Following roll bonding, the composite laminates were subjected to solution treatment followed by artificial aging. The solution treatment was conducted at 470 °C for holding times of 1, 4 and 8 h, which was immediately followed by an aging treatment at 120 °C for 24 h.

### 2.2. Microstructure Characterization

Metallographic samples were prepared by grinding and polishing. The grain size and shape of the samples subjected to different solution times were observed after the specimens were chemically etched at room temperature with Keller’s reagent (1 vol % HF, 1.5 vol % HCl, 2.5 vol % HNO_3_, and 96 vol % H_2_O) for 35 s. Microstructures of the composite laminates were observed using optical microscopy (OM) and scanning electron microscopy (SEM, Zeiss Ultra-55, Carl Zeiss, Jena, Germany) equipped with energy-dispersive X-ray spectrometry (EDS).

### 2.3. Mechanical Tests

The microhardness along the rolling direction of the laminates was tested in the as-rolled and solution-treated states using a Vickers microhardness tester (401-MVD, Wolpert Wilson Instruments, Esslingen, Germany) with a 25 g load for 10 s. For hardness testing, eight points (spaced 0.8 mm apart along the thickness direction) were selected within a 7075 layer and five points within a 1060 layer at the mid-thickness region of the composite sample, representing the hardness of the 7075 matrix and the 1060 layer, respectively.

**Figure 1:**
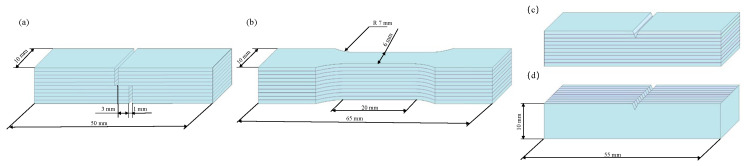
Schematic diagrams of the composite laminate specimens used in (**a**) tensile-shear test; (**b**) tensile test; (**c**,**d**) Charpy impact test.

Given that the composite laminates consist of nine 1060 layers, the one at the mid-height position was selected to evaluate the bonding strength of the interfacial region. The interfacial bonding strength of the composite laminates was evaluated using tensile-shear tests. The peak value of shear stress from the shear stress–displacement curves was defined as the shear strength of the laminates. The tensile-shear tests were carried out at room temperature with a crosshead speed of 1 mm/min. Figure 1a illustrates the tensile-shear specimen configuration used in this study.

After the heat treatment of the rolled laminates, tensile tests were performed on an electronic universal material testing machine at a strain rate of 1 × 10^−3^ s^−1^ at room temperature. Tensile tests of the as-received T6-tempered monolithic 7075 plates (7075-T6) were also conducted. All tensile tests were conducted in accordance with Chinese National Standard GB/T 228.1-2021 [18]. Tensile test specimens with a gauge length of 20 mm and a width of 6 mm were machined from the laminates, as shown in Figure 1b. Three-point bending tests were performed at room temperature using the same specimen geometry as the Charpy impact specimens, except that the notch was cut to a depth of 1 mm by using electrical discharge machining. The prepared specimens were in the crack-arrester orientation, and the crosshead speed was maintained at 1 mm/min throughout the bending tests. Tensile-shear, tension and bending tests were performed on a SANS CMT5105 electronic universal material testing machine (Sansi Yongheng Technology (Zhejiang) Co., Ltd., Ningbo, China).

V-notched Charpy impact specimens were cut with the dimensions of 10 × 10 × 55 mm^3^ (Figure 1c,d) using electrical discharge machining. A 2 mm deep V-notch was fabricated at the mid-length of the Charpy impact specimen. The sample preparation was in accordance with ASTM E23 [19]. The impact direction was vertical and parallel to the 7075/1060 interface. Tests were conducted on a SANS ZBC2452-C impact tester in both the crack-arrester and crack-divider orientations.

All samples for the mechanical property test were taken along the transverse direction (TD) of the laminates, and two coupons of each sample were tested.

## 3. Results and Discussion

### 3.1. Microstructures

**Figure 2:**
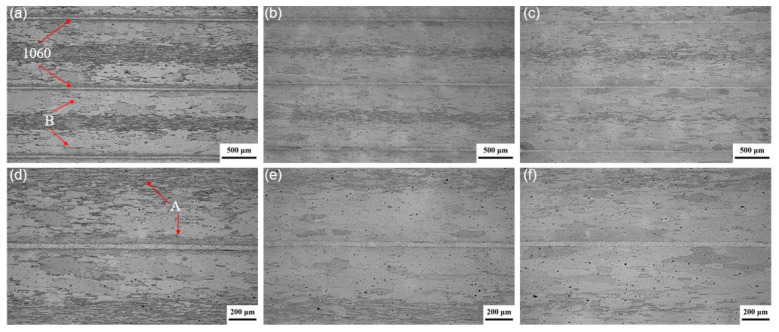
Optical micrographs of the composite laminates solution-treated at 470 °C for holding time of (**a**,**d**) 1 h; (**b**,**e**) 4 h; (**c**,**f**) 8 h.

Figure 2 shows optical micrographs of the interfacial region and the 7075 layer in the multilayered composite laminates after solution treatment at 470 °C for times ranging from 1 to 8 h. The 7075/1060 interfaces are distinct and macroscopically planar in all samples. Furthermore, no necking or fracture was observed in the 1060 layer, which is attributed to its good ductility. The absence of grains spanning the interface indicates that it acts as a strong barrier to grain boundary migration during solid solution treatment.

As shown in Figure 2a, the 7075 layer exhibits a microstructure consisting of a coarse recrystallized grain band around the 1060 layer, as shown by B in Figure 2a. Additionally, a large number of fine, elongated grains are distributed both adjacent to the 1060 layer and in the central region of the 7075 layer, as shown by A in Figure 2d. The 1060 layers are indicated by red arrows in Figure 2a. The coarse recrystallized grains observed within the 7075 layer can be attributed to the high rolling reduction employed in this study and the subsequent solution treatment, which induced recrystallization. The abnormally coarse recrystallized grains in region B can be attributed to the continuous diffusion of Zn and Mg elements from the 7075 layer into the 1060 layer during solution treatment. This resulted in a significant reduction in solute atoms in the 7075 near the 1060 layer, which otherwise exert a pronounced pinning effect on grain boundary migration.

Despite the continuous depletion of solute atoms in the 7075 layer, fine-grain bands adjacent to the 1060 layer are still present in the 7075 layer, as shown in Figure 2a,d. These grains are likely due to the fine oxide particles and other defects introduced into the 7075 surface layer by scratch brushing of 7075 surface prior to hot rolling. These particles may inhibit recrystallization and grain growth during subsequent solution treatment by pinning grain boundaries.

As shown in Figure 2, the thickness of the fine-grain band in the 7075 layer decreases as the solution time increases. After 8 h of solution treatment, fine grains are only observed in the mid-thickness region of the 7075 layer, while they are no longer visible near the 7075/1060 interface, as shown in Figure 2d–f. This indicates that grain growth has occurred in these fine-grain regions under prolonged solution treatment.

**Figure 3:**
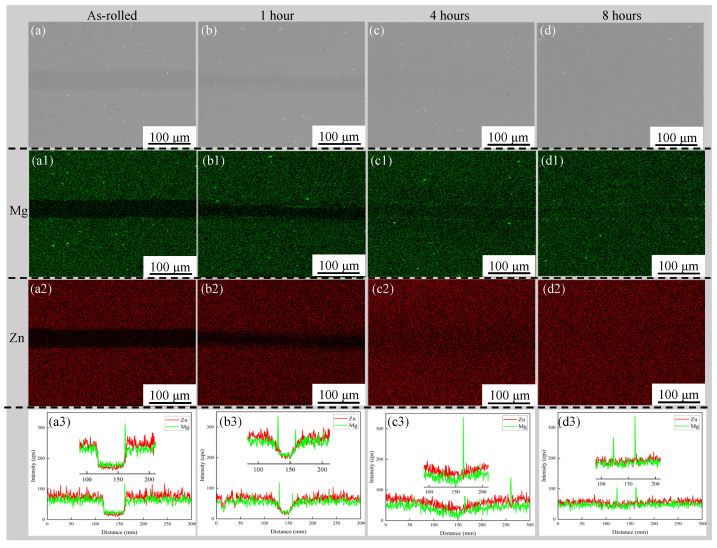
SEM micrographs and EDS results of the as-rolled (**a**–**a3**) and solution-treated composite laminates after holding at 470 °C for 1 h (**b**–**b3**); 4 h (**c**–**c3**) and 8 h (**d**–**d3**); (**a1**–**d1**) area distribution of alloying element Mg; (**a2**–**d2**) area distribution of alloying element Zn; (**a3**–**d3**) line scan profiles. The 1060 layer is located in the center of each micrograph.

Figure 3 shows the SEM micrographs and elements analysis of the as-rolled and solution-treated composite laminates under different solution holding times. Energy-dispersive X-ray spectrometry (EDS) mapping of Zn and Mg elements was performed, and the line scan results for Zn and Mg elements are also shown in Figure 3. It is observed that the 7075/1060 interfaces in the composite laminates subjected to different solution times became less distinct compared to those in the as-rolled condition. After 8 h of solution treatment, the interface between the 7075 layer and the 1060 layer became indistinguishable due to the significant diffusion of Zn and Mg elements into the 1060 layers.

The variation in Zn and Mg concentrations within the 1060 layer with increasing solution treatment time can also be observed from the line scan results. As shown in Figure 3(a3–d3), the line scan profiles demonstrate that the diffusion depth of Zn and Mg in the 1060 layer gradually increases with solution time. This may lead to enhanced solid solution strengthening of the 1060 layer with prolonged holding time. Line scan analysis reveals that the concentrations of magnesium and zinc in the 7075 layer decrease as the solution time increases.

Line scan results in Figure 3 indicate that in the as-rolled state, the concentration of Zn in the 7075 matrix is higher than that of Mg, whereas in the 1060 layer, the concentration of Mg exceeds that of Zn. This indicates a higher diffusion rate of magnesium compared to zinc in the as-rolled state. This may be attributed to the presence of an Al_2_O_3_ oxide film at the interface and the stronger oxidation affinity of Mg than Zn at the roll-bonding temperatures. After solid solution, the concentration of zinc gradually catches up with and eventually overtakes that of magnesium.

In the samples subjected to different solution holding times, an enrichment of the Mg element can always be observed at the 7075/1060 interface in the element distribution map (Figure 3(a1–d1)). A concentration peak of Mg at the interface is also observed in the line scan results of these samples (Figure 3(a3–d3)). It is hypothesized that this likely results from the formation of oxides between Mg and oxygen present at the 7075/1060 interfaces. The mapping results in Figure 3(a1–d1) also show that the Mg enrichment near the interface forms unevenly distributed particles.

### 3.2. Microhardness

**Figure 4:**
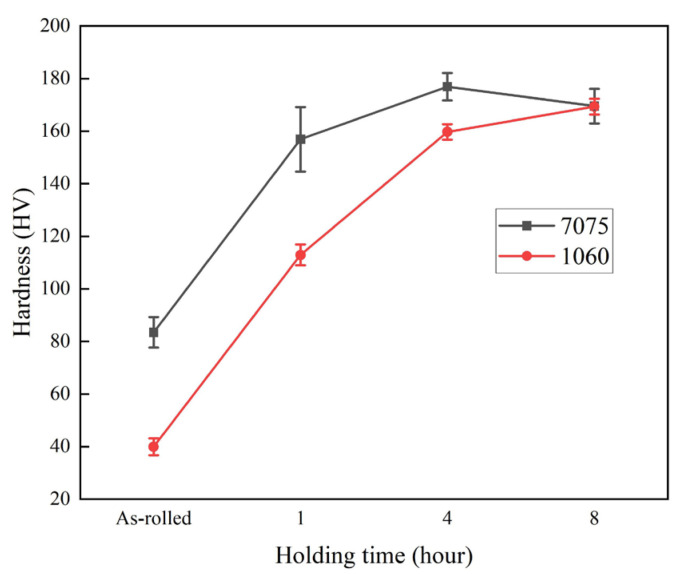
Microhardness profiles of the 7075 and 1060 layers in the composites under as-rolled and solution-treated conditions.

Figure 4 presents the microhardness profiles of the 7075 layer and the 1060 layer in the laminates in both the as-rolled and the solution-aged laminates. It can be observed that the hardness of both 7075 and 1060 layers increases with prolonged solution time during the initial stage of solution treatment. The 7075 layer achieved its maximum hardness after 4 h of solution treatment. The stabilization and slight decline in hardness of the 7075 layer between 4 and 8 h of solution treatment can be attributed to the softening of 7075, as evidenced by the increase in grain size of the 7075 layer (Figure 2). The hardness of the 1060 layer can be found to continue to increase with prolonged solution time until it reaches a level comparable to that of the 7075 layer after 8 h of solution treatment. The continuous increase in hardness of the 1060 layer is attributed to the sustained rise in solute concentration within the 1060 layer, which results in precipitation strengthening after aging treatment.

### 3.3. Interface Shear Strength

**Figure 5:**
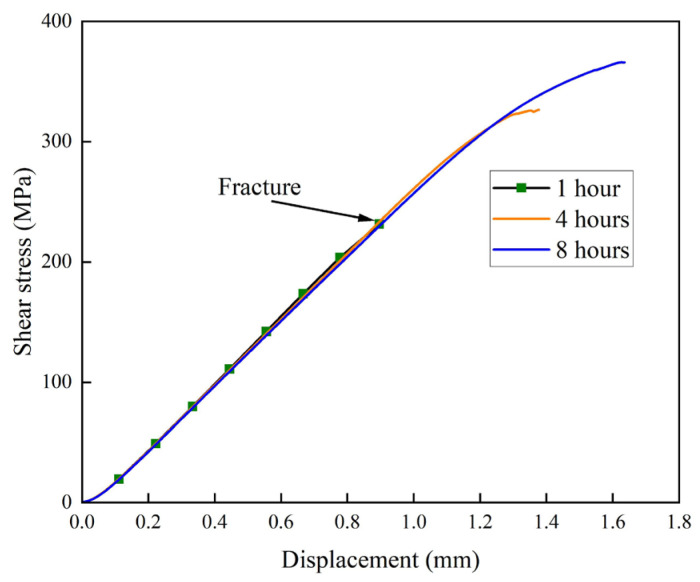
Shear stress–displacement curves of the composite laminates solution-treated at 470 °C for different holding times.

Figure 5 shows the shear stress–displacement curves of the multilayered composite laminates after holding at 470 °C for different times. As can be observed from the curves, the shear stress exhibits a continuous increase with the prolongation of solution treatment time, and the highest shear strength is 365.8 MPa in the sample with a holding time of 8 h. This indicates that increasing the solution treatment time can further enhance the interfacial bonding strength of the laminates. This corresponds to the trend of increasing elemental concentration with solution time presented in Figure 3. The increase in interfacial bonding strength is principally driven by the solid-solution and aging strengthening in the 1060 layer, as evidenced by the time-dependent hardness changes shown in Figure 4. It is also noted from Figure 5 that there is no further plastic deformation when the samples reach their ultimate shear stress.

This is in sharp contrast to those observed in the interface shear test in Cepeda-Jiménez’s research where much higher elongation-to-failure values have been obtained in certain 7075/1050 interfaces [20,21]. The rapid fracture following the maximum shear stress in the present laminates can be attributed to the limited plastic deformation permitted by the very thin 1060 layer. Figure 5 also shows that the fracture displacement increases with the holding time. This increase in both the shear strength and fracture displacement indicates an increase in the area under the shear stress–displacement curves, which is responsible for the improved toughness of the interface after longer solution treatment. This improvement of interface mechanical properties after prolonged solid solution treatment has also been observed by Zhang et al. in their 7050 aluminum alloy clad plates [22].

**Figure 6:**
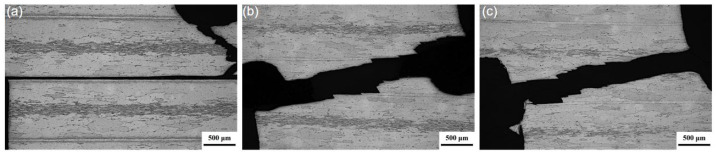
Surface fracture morphology of tensile-shear laminates solution-treated at 470 °C for holding times of (**a**) 1 h; (**b**) 4 h; (**c**) 8 h.

Figure 6 shows the surface fracture morphologies of the tensile-shear test laminates solution-treated at 470 °C for different holding times. In each image, the central light-colored region corresponds to the 1060 layer. The two machining notches of the sample are located at the lower left and upper right positions of each image. At all solution times, the laminates exhibited a mixed fracture behavior characterized by interfacial failure at the 7075/1060 interface and fracture within the 7075 layer. As observed in the sample solution-treated for 1 h (Figure 6a), the crack path deviated from the intended 1060 interfacial region between the two notches and instead propagated along another 1060 interfacial zone. Further observation reveals that the crack path is along the 7075/1060 interface rather than through the interior of the 1060 layer. These suggest that the crack propagation resistance of the 1060 layer is not only higher than that of the 7075/1060 interface region but also surpasses that of the 7075 layer. With the solution treatment time prolonged to 4 and 8 h, the crack propagated predominantly along the 7075 layer with occasional deviation along the 7075/1060 interface. As shown in Figure 2, the proportion of abnormally coarsened grains in the 7075 layer continuously increases with prolonged solution time. This corresponds to the transition in the tensile-shear fracture path from propagation along the 7075/1060 interface to primarily through the 7075 matrix. This indicates that the abnormally coarsened grains possess lower resistance to crack propagation.

### 3.4. Tensile Properties

**Figure 7:**
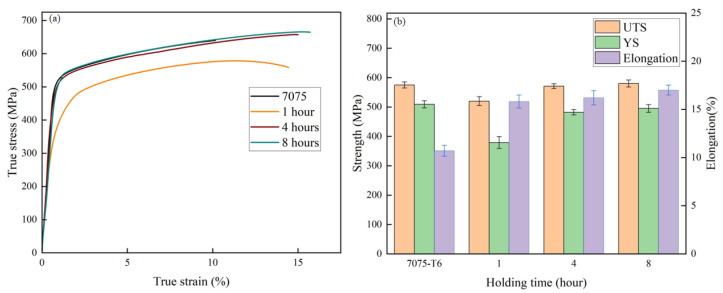
True stress–strain curves (**a**) and yield strength, ultimate tensile strength and elongations (**b**) of the laminates solution-treated at 470 °C for different holding times.

To investigate the uniaxial deformation behavior of the multilayered composite laminates under different solution times, tensile tests were conducted and the true stress–strain curves were obtained, as shown in Figure 7a. In contrast to the laminates solution-treated for 1 h, both the laminates with other solution times and the commercially purchased 7075 monolithic plate exhibited continuous work hardening. Compared to the laminate, the monolithic 7075 plate exhibits the lowest elongation. Figure 7b illustrates the variation in yield strength, tensile strength, and elongation derived from the tensile tests. For comparison, the figure also includes data from a commercially purchased 7075 monolithic plate. As shown in the figure, the sample with 1 h holding time exhibits the lowest tensile strength of 519.9 MPa. When the solution time is extended to 4 h, the tensile strength of the composite laminate increases to 571.7 MPa, which is nearly comparable to that of the T6-tempered 7075 monolithic plate. With a further increase in holding time to 8 h, the composite laminate achieves the highest ultimate tensile strength and elongation among all samples, reaching 580.1 MPa and 17.1%, respectively. It is noteworthy that the elongation of the composite laminate with an 8 h solid solution treatment achieved an increase of nearly 60% compared to the as-purchased 7075 plate. Such a dual increase in strength and elongation with holding time may be caused by extra heterogeneous deformation-induced (HDI) hardening due to the introduction of numerous interfaces between hard 7075 and soft 1060 layers [23,24,25].

**Figure 8:**
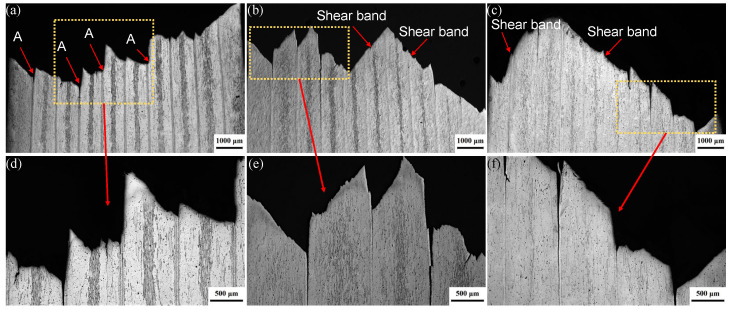
Optical micrographs of the rolling direction (RD)–normal direction (ND) sections of the tensile-fractured laminates solution-treated at 470 °C for holding times of (**a**) 1 h; (**b**) 4 h; (**c**) 8 h. (**d**–**f**) detailed images of the region in the vicinity of the fracture surface.

Figure 8 shows surface morphology on through-thickness RD-ND sections of the laminates solution-treated at 470 °C for different holding times to clarify the micro-mechanisms associated with the fracture morphology. Delamination cracks were observed near the fracture surface for the laminates. Further characterization near the fracture surface indicated that these cracks propagate predominantly along the 7075/1060 interfaces, as shown in Figure 8d–f. This finding is in agreement with the experimental observations of crack paths from the tensile-shear experiments. From the observation, it is also noted that the height difference between adjacent 7075 layers was most pronounced in the sample solution-treated for 1 h (as shown by A in Figure 8a), indicating relatively low interfacial bonding strength in this sample. When the solution time was increased to 4 h, shear fracture along several local 7075 layers was observed on the fracture surface, as shown in Figure 8b. In the sample solution-treated for 8 h, shear fracture occurred mainly along two distinct shear fracture angles, as shown in Figure 8c. These observations further indicate that the interfacial bonding strength increases with prolonged solution treatment time.

### 3.5. Three-Point Bending Test

**Figure 9:**
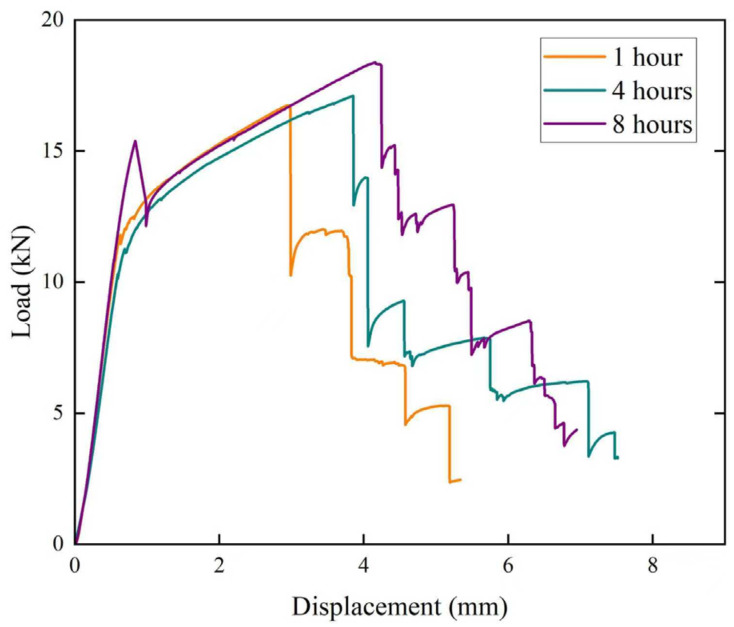
Load–displacement curves of the three-point bending test of the composite laminates solution-treated at 470 °C for different holding times.

To investigate the crack propagation behavior of the composite laminates in the crack-arrester orientation, notched composite laminates were subjected to three-point bending tests to observe the influence of the thin 1060 layers on the crack propagation. Figure 9 shows the representative load–displacement curves of the composite laminates solution-treated at 470 °C for 1, 4 and 8 h. For all samples, the load–displacement curves exhibit an initial continuous increase in load up to the maximum load, which is followed by a stepwise drop of load. A closer examination reveals that for all samples, the curves exhibit a load drop in the initial rising stage, which is followed by a gradual reloading prior to reaching the global maximum load. Since the machined notch was located within the first 7075 layer, this initial load drop is attributed to the rapid propagation of a crack from the notch within this first 7075 layer. This phenomenon is most pronounced in the sample with a holding time of 8 h. The subsequent reloading indicates that the advance of the main crack is effectively impeded at the first 7075/1060 interface. After the global maximum load of the entire load–displacement curve, all the samples with various holding times are generally characterized by a rapid load drop followed by a load stabilization plateau. This rapid-drop-and-plateau pattern persists until final fracture. A comparison of the bending curves shows that the maximum bending load consistently increases with holding time, reaching a peak value of 18.3 kN after holding at 470 °C for 8 h. It is also noted from Figure 9 that not only the maximum load but also the displacement corresponding to this maximum load demonstrates an increasing trend with extended solution treatment time. This demonstrates that the crack growth resistance of the material was enhanced with prolonged solution treatment time.

**Figure 10:**
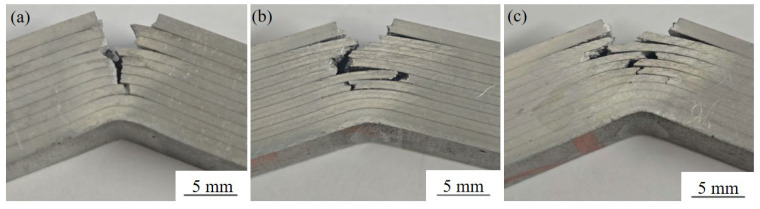
Surface fracture morphologies of the three-point bending test specimens solution-treated at 470 °C for holding times of (**a**) 1 h; (**b**) 4 h; (**c**) 8 h.

The fracture process, characterized by the sequential main crack propagation, delamination, re-nucleation and growth of subsequent cracks, is evident from the surface observation of the bending specimens, as shown in Figure 10. Observations of the main crack on the sample surface and the 1060 layer reveal that the rapid load drops correspond to the propagation of the main crack through the 7075 layers, whereas the plateau region corresponds to delamination occurring at the 7075/1060 interfaces, as shown in Figure 10. Further observation indicates that once delamination starts along the interface, the propagation of the main crack in the 7075 layer is suppressed, and load decreases do not occur until crack re-nucleation occurs in the next 7075 layer.

In Figure 10, fracture through delamination can be observed, and more extensive delamination can be noted in samples with longer holding times. Generally, any increase in the bending toughness of a laminate is due to intrinsic and extrinsic mechanisms of fracture. Intrinsic mechanisms originate from the microstructure of the constituent materials, and interface delamination is a typical extrinsic mechanism. From Figure 9, a relatively longer plateau region is observed in laminates with longer solution times of 4 and 8 h. This indicates that more energy is consumed on 7075/1060 interfacial cracking before re-nucleation occurs in the subsequent 7075 layer.

Chen’s group investigated the bending fracture behavior of AA1100/AA7075 laminated metal composites [26]. They found that as the number of layers increases, the 1280-layer specimen exhibited a lower toughness than other LMCs with fewer layers. This is likely attributed to a degradation in the toughening effect from interfacial debonding, resulting from the increased interfacial bonding strength with a higher number of ARB passes. From these investigations, an appropriate interfacial bonding strength plays a critical role in the toughening of composite laminates.

In addition to the delamination resistance of the 1060 layer, the toughness of the 7075 layer in the present composite laminates is also crucial for deciding the fracture resistance of the laminated samples. As a general observation, it can be stated that the dissolution of constituent phases and strengthening phases in the 7075 matrix is enhanced by higher solution treatment temperatures and longer duration, which contributes to a more homogeneous microstructure and, consequently, improved fracture toughness. The desirable combination of strength and fracture toughness at elevated solution temperatures and extended holding times has been reported in other age-hardenable aluminum alloys [27,28]. Based on the extent of delamination (Figure 10) and the areas under the bending load–displacement curves (Figure 9), it can be concluded that the toughness of the composite laminates increases with prolonged solution time within the range investigated.

### 3.6. Charpy Impact Test

**Figure 11:**
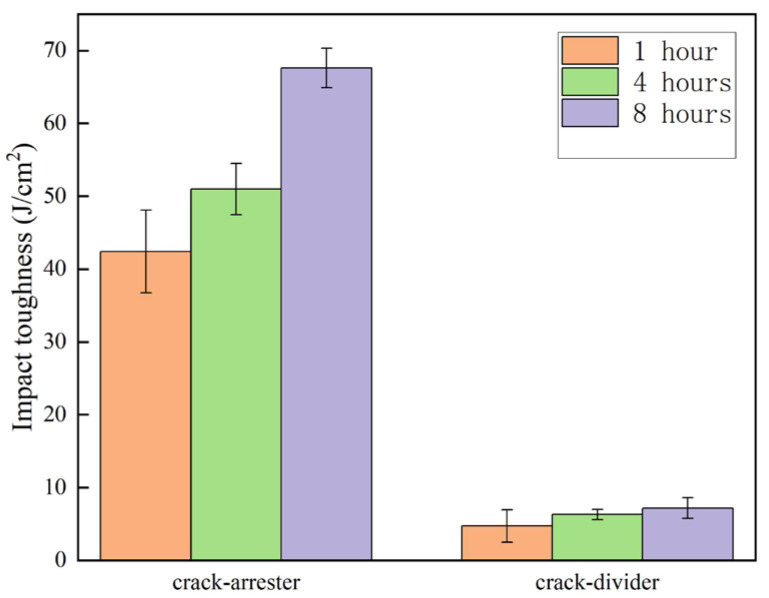
Impact toughness of the composite laminates solution-treated at 470 °C for different holding times in both crack-arrester and crack-divider orientation.

**Figure 12:**
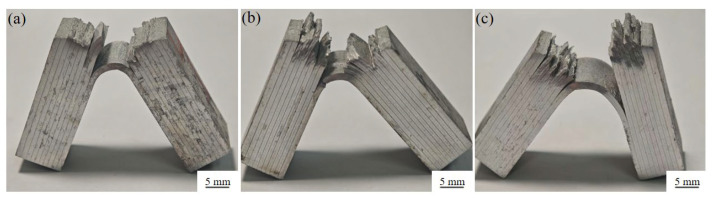
Surface fracture morphologies of notch-impact-tested composite laminates solution-treated at 470 °C for holding time of (**a**) 1 h; (**b**) 4 h; (**c**) 8 h.

The Charpy impact tests were performed on the multilayered composites in the crack-arrester and crack-divider orientation at room temperature. Figure 11 shows that the average impact toughness of the samples with a holding time of 1, 4 and 8 h in the crack-arrester orientation is 42.4, 50.9 and 67.6 J/cm^2^, respectively, whereas in the crack-divider orientation, the corresponding values are 4.7, 6.3 and 7.2 J/cm^2^, respectively. It can be observed that the impact toughness increases with prolonged holding time in both crack orientations. It is also noted that the impact toughness of the sample in the crack-arrester orientation is much higher than that of the crack-divider orientation, which is consistent with the results in [29,30]. According to Figure 11, the impact toughness in the crack-arrester orientation is about eight times higher than that in the crack-divider orientation. The inferior impact toughness of the latter is attributed to the absence of interface-related arresting mechanisms. Figure 12 shows the optical images of the fractured Charpy impact samples of the multilayered composite laminates with different holding times. More tortuous crack paths formed in samples with a holding time of 4 h or longer, and apparent delamination occurred at most of the interfaces in these laminates.

This observed delamination toughness is responsible for the significant Charpy impact toughness in Figure 11. Similar delamination toughening has been observed in other laminated materials [31,32,33,34,35,36].

The similar fracture paths and degrees of delamination observed in the present multilayered composite laminates during both bending and Charpy impact tests indicate that the active plastic deformation and interface delamination mechanisms remain unchanged. This similarity further demonstrates the absence of a significant strain rate effect on the fracture behavior between the two testing methods.

**Figure 13:**
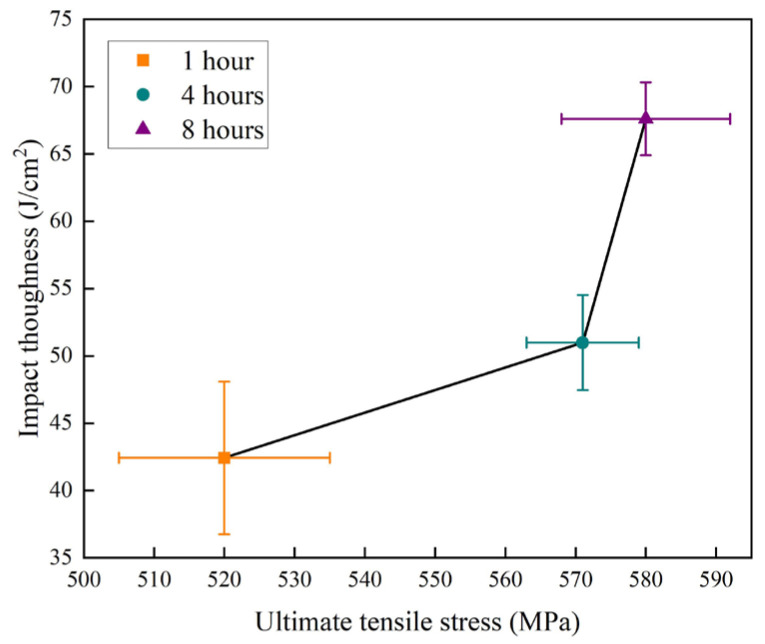
Relationship between ultimate tensile strength and impact toughness of the composite laminates solution-treated at 470 °C for 1, 4 and 8 h.

Based on the preceding tensile and Charpy impact results, Figure 13 summarizes the relationship between the ultimate tensile strength and impact toughness of the composite laminates under different solution holding times. The figure reveals a concurrent improvement in both strength and impact toughness with prolonged solution time. From the previous research [27,28], it can be stated that the extended solution holding time facilitates the dissolution of residual phases within the 7075 matrix, which enhances the intrinsic toughness and tensile strength. Although the grain size of the 7075 layers gradually increased with extended solution time (Figure 2), an effect that would reduce grain boundary strengthening and thus material toughness and strength, the prolonged solution time also enhanced the potential for precipitation strengthening. The overall effect of holding time at 470 °C is that both the impact toughness and tensile properties of the composite laminate improve with increasing duration.

## 4. Discussion

In this experimental study, a 7075 composite laminates was fabricated using a 1060 layer as the interlayer. Subsequent solution and aging treatments enhanced the interfacial bonding strength and increased the solid solution strengthening degree of the 1060 layer. Based on the EDS mapping and line scan results of the interfacial regions around the 1060 layer, both the extent and the penetration depth of Zn and Mg diffusion increase with prolonged solution time. Notably, Mg segregation was observed at the 7075/1060 interface in composites subjected to different solution treatments (Figure 3). Yue et al. reported a similar segregation of MgO particles at the interfaces of roll-bonded and subsequently solution-treated 7050/pure Al and 7A62/pure Al composites [37]. It is therefore hypothesized that the Mg segregation observed in the present study may likewise originate from the diffusion of Mg from the 7075 layer to the 7075/1060 interface during solution treatment, where it reacts with pre-existing oxides to form MgO. It should be noted that in Yue’s work, the MgO particles were non-uniformly and sporadically distributed, and their specific effect on interfacial bonding strength was not further examined. Although the solution-treated composites in the present experiment exhibited significant grain growth in certain regions of the 7075 matrix with increasing solution time, the research by Ma et al. on strengthening mechanisms in aluminum alloys indicates that the contribution of grain boundary strengthening (the Hall–Petch effect) to yield strength is significantly smaller than that of precipitation strengthening [38]. The mechanical properties of the solution-treated composites may be degraded by both grain coarsening in the 7075 layer and the presence of non-uniformly dispersed fine particles at the 7075/1060 interface. The formation mechanism of these brittle Mg-containing oxides at the 7075/1060 interface, their evolution during solution and aging treatments, and the influence of their size and distribution on interfacial bonding strength remain unclear. Addressing these questions therefore represents a distinct and worthwhile direction for future research.

Considering that both the hardness of the 1060 layer and the bonding strength of the 7075/1060 interface increase with prolonged solution time, these factors are conducive to the performance improvement of the composite laminate. Furthermore, the multilayer composite laminate in this study is a heterogeneous layered material, where beneficial hetero-deformation induced (HDI) strengthening, and strain hardening can be generated through the accumulation of geometrically necessary dislocations (GNDs). Therefore, by rational design of the layer structure and utilization of the mechanical incompatibility between hard and soft layers [24], laminated composites with an excellent strength–ductility synergy can be fabricated.

This study primarily correlated the macroscopic mechanical properties (e.g., tensile and flexural strength) with the microstructures observed via optical and scanning electron microscopy. However, it lacks direct microscopic evidence regarding the nanoscale interfacial structure, the localized deformation mechanisms at crack tips, and the precise fracture modes. Possible measures to avoid them could be using electron backscatter diffraction (EBSD) to analyze the grain orientation, strain localization, and recrystallization behavior in the vicinity of bending or impact crack paths. Potential points for future research in this area could involve employing in situ bending/impact stages within transmission and scanning electron microscopes (TEM/SEM), which would enable the direct observation of nanoscale interfacial precipitates, dislocation motion, and the nucleation and propagation of micro-cracks.

## 5. Conclusions

In this study, multilayered composite laminates with alternating 7075 and 1060 layers were successfully fabricated by roll bonding with a total reduction of 50%. The composite laminates underwent solution treatment at 470 °C for various holding times followed by aging treatment to enhance their strength. The main conclusions derived from this study are as follows:

(1) As the holding time increases at 470 °C, the concentrations of Zn and Mg elements dissolved in the 1060 layer increase, resulting in an enhanced solid solution strengthening effect. This leads to a simultaneous increase in the shear strength and shear displacement of the bonding interface with prolonged holding time, thereby achieving high interface toughness.
(2) There is a dual increase in the tensile strength and elongation as the solution holding time increases. After a solution treatment of 8 h, the composite laminate exhibits higher tensile strength and elongation compared to the as-received T6-tempered 7075 monolithic plate. Notably, the elongation exhibited an increase of nearly 60% compared to that of the as-received T6-tempered 7075 monolithic plate.
(3) The tensile, Charpy impact and bending properties are all positively correlated with the solution holding time. As the solution holding time increases, both interfacial delamination and the degree of main crack deflection increase in the Charpy impact and bending tests, which in turn lead to an improvement in both the laminate’s impact and bending properties.

## Figures and Tables

**Table 1:** Chemical composition of the as-received 7075 aluminum alloy (wt. %).

	Zn	Mg	Cu	Fe	Mn	Cr	Ti	Zr	Si	Al
7075	5.9	2.7	1.6	0.31	0.17	0.20	0.03	0.08	0.14	Bal.
1060	0.03	0.01	0.02	0.15	0.01	-	0.01	-	0.13	Bal.

**Table 2:** Mechanical properties of the as-received 7075 aluminum alloy.

	UTS (MPa)	YS (MPa)	Elongation (Pct.)
7075-T6	575 ± 25 *	509 ± 30 *	10.7 ± 1.4 *

* Values represent mean ± half-width of the 95% confidence interval (n = 3).

## Data Availability

The original contributions presented in this study are included in the article. Further inquiries can be directed to the corresponding author.
